# Pharmacokinetics and pharmacodynamics of the denosumab biosimilar FKS518 and reference denosumab in healthy subjects: the Lumiade-1 study

**DOI:** 10.1093/jbmrpl/ziaf176

**Published:** 2025-11-07

**Authors:** Anna Dryja, Mateusz Cieślak, Piotr Sobieraj, Peter Szeles, Adriano L S de Souza, Joelle Monnet

**Affiliations:** MTZ Clinical Research powered by PRATIA, 02–172 Warsaw, Poland; MTZ Clinical Research powered by PRATIA, 02–172 Warsaw, Poland; MTZ Clinical Research powered by PRATIA, 02–172 Warsaw, Poland; Department of Clinical Development, Fresenius Kabi SwissBioSim, CH–1262 Eysins, Switzerland; Department of Clinical Development, Fresenius Kabi SwissBioSim, CH–1262 Eysins, Switzerland; Department of Clinical Development, Fresenius Kabi SwissBioSim, CH–1262 Eysins, Switzerland

**Keywords:** biosimilar, denosumab, equivalence, FKS518, pharmacokinetics, pharmacodynamics, tolerability, immunogenicity

## Abstract

Denosumab is a fully human monoclonal antibody that increases BMD, inhibits bone resorption and reduces fracture risk. This double-blind, randomized, parallel group study aimed to demonstrate the pharmacokinetic (PK) equivalence and compare the pharmacodynamic (PD), safety and immunogenicity profiles of the proposed denosumab biosimilar FKS518 vs the originator (reference) denosumab. Healthy males (28-55 yr) were randomized to a single 60 mg s.c. injection of FKS518 or the reference denosumab (Prolia) and were followed for 40 wk after drug injection. The primary endpoints were area under the concentration-time curve (AUC) from time zero to infinity, AUC from time zero to the last quantifiable concentration, and maximum observed serum concentration. A total of 213 subjects were injected. Pharmacokinetic equivalence was demonstrated as the 90% CIs for the geometric least squares means ratio FKS518/reference denosumab for the three primary PK parameters were fully contained within the predefined bioequivalence limits. Secondary PK, PD, safety, and local tolerability endpoints also supported the similarity of FKS518 and reference denosumab. No anti-drug antibodies were detected in either treatment group. These results demonstrate that FKS518 is equivalent to originator denosumab with respect to PK profile.

## Graphical Abstract

**Graphical Abstract:**
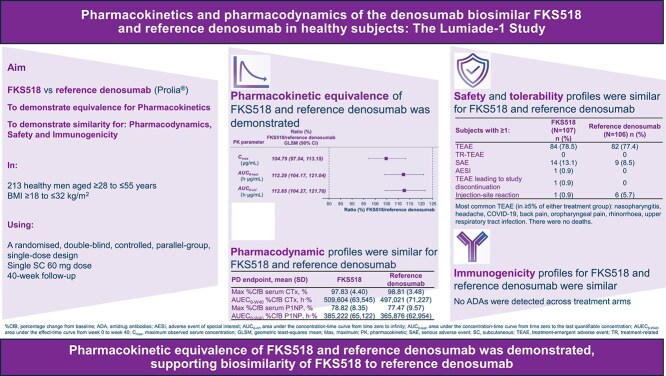

## Introduction

Denosumab is a fully human monoclonal antibody of IgG2 subtype that inhibits RANKL.^1^ This ligand is essential to the formation, activation and survival of osteoclasts. Its production is increased when estrogen levels are low, such as after menopause or hormone ablation therapy, leading to increased bone resorption.^2^ Denosumab inhibits high bone turnover (which is associated with reduced BMD and adversely altered bone architecture and porosity), increases BMD and consequently reduces fracture risk in target populations, and it is generally considered a safe treatment, even when administered long-term.^2–6^ While BMD is widely used to diagnose osteoporosis and predict fracture risk, changes in bone turnover markers, such as CTx and P1NP indicate bone resorption and bone formation, respectively, and their suppression is also associated with reduced fracture risk.^7^

Denosumab is marketed in the European Union (EU) and United States (US) as Prolia and Xgeva. Prolia (denosumab 60 mg) is indicated for the treatment of osteoporosis in postmenopausal women and in men at increased risk of fractures. It is also approved as treatment of bone loss associated with hormone ablation in men with prostate cancer at increased risk of fractures, in adult patients with long-term systemic glucocorticoid therapy at increased risk of fractures and, in the US, in women at high risk for fractures receiving adjuvant aromatase inhibitor therapy for breast cancer.^1^^,^^8^ Xgeva (denosumab 120 mg) is indicated for the treatment of giant cell tumor of bone, prevention of skeletal-related events in patients advanced malignancies involving bone and, in the US, treatment of hypercalcemia of malignancy refractory to bisphosphonates.^9^^,^^10^ The mechanism of action of denosumab is the same across all currently approved indications.^1^^,^^8–10^

In common with other biologic agents, denosumab is a large, complex molecule.^1^^,^^8–10^ Biosimilar medicines can be approved after completing a rigorous stepwise research exercise to establish similarity between the biosimilar and the reference (originator) biologic that includes physiochemical and biological characterization, as well as comparisons of pharmacokinetic (PK), pharmacodynamic (PD), efficacy, safety, and immunogenicity clinical properties of the 2 products.^11–14^ Biosimilars are typically cheaper to develop than the originator product, which can lead to cost savings and improved access to treatment, benefitting patients, and healthcare systems.^15^^,^^16^

FKS518 was developed as a biosimilar to the originator denosumab Prolia and Xgeva. Analytical and functional similarity has been demonstrated between FKS518 and the originator denosumab. Biosimilarity on the clinical level was to be demonstrated by conducting 2 clinical studies. The current study (EudraCT Number: 2020-004842-13; Lumiade-1) aimed to demonstrate PK equivalence and to compare the PD, safety, tolerability, and immunogenicity profile of FKS518 with US-licensed Prolia (reference denosumab) in healthy male subjects. Pharmacodynamic endpoints evaluated the association of denosumab treatment with bone resorption (CTx) and bone formation (P1NP) biomarkers. Therapeutic equivalence in women with postmenopausal osteoporosis was investigated in LUMIADE 3 (EudraCT Number: 2020-004422-31), the results of which are presented separately.^17^ Both studies were designed in accordance with regulatory guidance from the U.S. Food and Drug Administration and European Medicines Agency.

## Materials and methods

### Study design

This was a double-blind, randomized, two-arm, parallel group study of FKS518 and reference denosumab in healthy male subjects enrolled at a single investigative site in Poland. Eligible subjects were randomized on day −1 to a single s.c. injection of FKS518 60 mg or reference denosumab 60 mg, to be administered into the abdomen on day 1 after an overnight fast that lasted until the 4-h post-dose PK and PD blood samples had been collected. Randomization was performed by an Interactive Response Technology system, in permuted blocks, and subjects were stratified by weight (WT) (50 ≤ WT ≤ 70 kg vs 70 < WT ≤ 110 kg). The subjects, investigators, and the sponsor were blinded to the product administered until the database lock following the end of the study.

The study duration was 44 wk, including a screening period of ≤4 wk, administration of study drug on day 1, and a follow-up period of 40 wk, with 1 wk of confinement in the clinic (days −1 to 6) and 16 ambulatory visits up to day 274. Blood samples for PK, PD, and immunogenicity testing were collected predose (0 h) and at scheduled time points up to day 274 (see Supplementary material for details). Blood samples for PD endpoints were collected in the morning (between 8 am and 12 pm), after an overnight fast. Pharmacokinetic, PD (CTx and P1NP), and immunogenicity sample analyses were performed using validated methods by a qualified laboratory. For the PK assay, detection and capture antibodies were the denosumab antibody AbD26781_hIgG1: Bio-Rad Cat# HCA283 (RRID:AB_3712665) and the denosumab antibody AbD26295_hIgG1: Bio-Rad Cat# HCA280 (RRID:AB_3712666). The NAb assay used RANKL as the capture reagent (Cell Sciences Cat# CRR100-AF, RRID:AB_3712736). The CTx assay used was from Immunodiagnostic Systems (Cat# AC-02F1, RRID:AB_2923399), and the P1NP assay was the P1NP Cobas assay (Elecsys total P1NP kit; Roche Cat# 03141071190, RRID:AB_2782967).

### Participants

Eligible subjects were healthy males aged ≥28 to ≤55 yr, with a body WT of 50 ≤ WT ≤ 110 kg and BMI of 18.0 ≤ BMI ≤ 32.0 kg/m^2^, and with clinically acceptable physical examinations and laboratory tests. Subjects were either surgically sterile or had to use a condom in addition to having their female partner of childbearing potential use another form of contraception during the study period.

Exclusion criteria included known or suspected hypersensitivity to denosumab or to any formulation component, comparable drugs, or latex; osteonecrosis of the jaw or risk factors for osteonecrosis of the jaw; evidence of hypocalcemia or hypercalcemia; known vitamin D deficiency; renal impairment; current or history of primary or secondary immunodeficiency; severe or recurrent herpes zoster or any opportunistic invasive infection within 6 mo before screening; frequent chronic or recurrent infections; positivity for human immunodeficiency virus subtype 1 or 2 or hepatitis C virus, or evidence of acute or chronic hepatitis B infection; serious infection within 8 wk before randomization; treatment with oral antibiotics and/or antifungal drugs within 14 d prior to screening; noteworthy surgical intervention within 8 wk before the study or a scheduled surgical procedure during the study; a history of or current, clinically significant alcohol or drug abuse or an inability to refrain from alcohol consumption from 48 h before denosumab administration and during confinement in the clinical site; prior use of denosumab or any other drug affecting bone metabolism; or vigorous exercise within 72 h of denosumab administration (verified by creatine phosphokinase blood levels).

The study was conducted during the COVID-19 pandemic, necessitating local site-specific COVID-19-related procedures to prevent infection during the study. Only subjects with a negative SARS-CoV-2 PCR test were allowed to participate in the study. Subjects could not have had a COVID-19 vaccine within 4 wk prior to randomization or an ongoing multidose vaccination regimen at the time of screening.

### Ethics

The study was conducted with written and dated approval from the locally competent Independent Ethics Committee, and in accordance with the Guidance on Good Clinical Practice International Council for Harmonization Harmonized Tripartite Guideline E6 (R2), requirements for the conduct of clinical studies as provided in the EU Regulation 536/2014, the general guidelines indicated in the Declaration of Helsinki and all applicable regulatory requirements.

All subjects provided written informed consent before any study-related activities were performed.

### Study endpoints

The primary objective of the study was to demonstrate PK equivalence between FKS518 and reference denosumab based on 3 primary PK endpoints: area under the concentration-time curve from time zero to infinity (AUC_0-inf_ calculated as AUC_0-last_ + C_last_/𝜆𝑧, where C_last_ was the last measurable serum concentration and 𝜆𝑧 was the terminal elimination rate constant), area under the concentration-time curve from time zero to the last quantifiable concentration (AUC_0-last_), and maximum observed serum concentration (*C*_max_). Secondary PK endpoints were time to reach peak serum concentration (*t*_max_), terminal elimination half-life (*t*_1/2_), volume of distribution during terminal phase (Vz/F), and apparent total clearance (CL/F).

Pharmacodynamic endpoints were area under the effect-time curve (AUEC) from week 0 to week 40 (AUEC_0-W40_) for percentage change from baseline (%CfB) in serum CTx and P1NP, %CfB at all time points postdose in serum CTx and P1NP, and maximum %CfB in serum CTx and P1NP. To allow assessment of the recovery of serum CTx and P1NP toward baseline levels, and to therefore capture a near complete PD response profile and aid identification of potential minor differences in potency or exposure, sampling for PD assessments was performed for 40 wk (to day 274). Pharmacokinetic sampling was also conducted for 40 wk (to day 274) to enable consistent evaluation of PK/PD correlations throughout the study duration.

Tolerability and safety endpoints were based on treatment-emergent adverse events (TEAEs) that were coded using MedDRA, Version 24.0. The intensity or severity of TEAEs was graded by the investigator using the National Cancer Institute-Common Terminology Criteria for Adverse Events (CTCAE), Version 5.0. Serious TEAEs (SAEs), injection-site reactions (ISRs; local tolerability), adverse events (AEs) of special interest (AESI; drug-related hypersensitivity/allergic reactions [CTCAE Grade ≥ 3 or reported as SAEs]), AEs leading to study withdrawal and clinically significant laboratory findings and vital signs (blood pressure, respiratory rate, pulse rate, or temperature) and 12-lead ECG abnormalities were reported. Confirmed COVID-19 infections were considered an “other medially important event” and reported as SAEs regardless of severity or meeting other seriousness criteria, given the limited knowledge of the infection at the time of the study. Investigators assessed the causal relationship of all TEAEs to denosumab administration. Secondary immunogenicity endpoints were incidence of anti-drug antibodies (ADAs), ADA titers, and incidence of neutralizing antibodies (NAbs).

An exploratory objective was to explore the exposure-response relationship between %CfB in serum CTx and P1NP biomarker and denosumab concentrations.

### Statistical analysis

A total of 214 healthy male subjects (107 per group) were planned to be enrolled, to target a minimum of 170 evaluable subjects in the PK Analysis Set, assuming a 20% drop-out rate. A total of 170 subjects would provide 90% power to demonstrate bioequivalence between the 2 treatments for the PK primary endpoints with a bioequivalence interval of [80.00%, 125.00%] and a type I error rate of 5%, assuming a maximum 5% difference between treatment groups on the geometric least squares mean ratio (GMR), and a coefficient of variation not larger than 40% for the PK primary endpoints.

The PK Analysis Set was used for PK evaluations, and included all subjects who received a complete dose of study drug, had no important protocol deviations affecting PK assessments and had enough bioanalytical assessments to calculate reliable estimates of at least 1 PK parameter. Pharmacokinetic parameters were determined using standard noncompartmental analysis with Phoenix WinNonLin (Version 8.3.4, Certara, L.P.) and statistical analyses were conducted with SAS v.9.4. Pharmacokinetic AUCs were computed using the linear up/log down method using all serum concentration results available for a given subject. In-between missing concentrations or concentrations below the limit of quantification were interpolated using this same method. Natural log-transformed PK primary endpoints (ie, AUC_0-inf_, AUC_0-last_, and *C*_max_) were analyzed using an ANOVA model with treatment and WT strata (50 ≤ WT ≤ 70 kg vs 70 < WT ≤ 110 kg) as fixed effects. For the comparison of primary endpoints, the 90% CIs for the GMR were derived by exponentiating the 90% CI obtained for the difference between the 2 treatments least-square (LS) means resulting from the analysis of the log-transformed PK primary endpoints. If the 90% CIs for the GMR of all PK primary endpoints were entirely within the 80.00%-125.00% equivalence margins, then PK equivalence between the 2 treatments could be declared. The secondary PK endpoints were summarized descriptively as median or mean with SD. Subgroup analysis was performed by WT strata.

The PD Analysis Set included all subjects who received a complete dose of study drug, had no important protocol deviations affecting PD assessments and had enough bioanalytical assessments, including a baseline concentration value, to calculate reliable estimates of at least 1 PD parameter. Pharmacodynamic AUECs were computed using the trapezoidal method. In-between missing concentrations or concentrations below the limit of quantification were interpolated using this same method. All PD endpoints were summarized using descriptive statistics.

The Safety Analysis Set included all subjects who received any dose of the study drug and was used for safety and immunogenicity analyses. Subjects were analyzed according to the actual treatment received. TEAEs were presented by system organ class (SOC) and preferred term (PT) in frequency tables, with subjects with multiple AEs counted only once within each PT and SOC. Serious TEAEs and AESIs were summarized in a similar manner and ISRs were listed and summarized descriptively. Immunogenicity endpoints (ADA incidence, ADA titres, and NAb incidence) were summarized descriptively.

## Results

**Figure 1:**
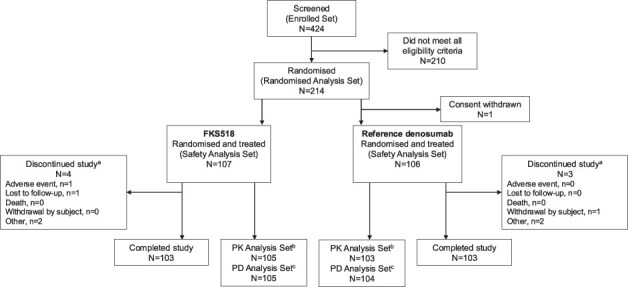
Study disposition. ^a^No study discontinuations were related to COVID-19. ^b^Excluded subjects had insufficient serum concentrations assessments to calculate at least one reliable PK parameter. ^c^Excluded subjects did not achieve maximal PD inhibition. Abbreviations: PD, pharmacodynamic; PK, pharmacokinetic.

The study was conducted between May 6, 2021, when the first subject was enrolled, and September 2, 2022, when the last subject completed his last visit. Of the 424 subjects screened, 214 subjects were randomized to receive FKS518 or reference denosumab. Of these, 1 subject who had been randomized to receive reference denosumab withdrew his consent prior to dosing. Therefore, 213 subjects received one dose of denosumab 60 mg (FKS518, *n* = 107; reference denosumab, *n* = 106) and all subjects received the full dose. A total of 206 subjects (96.3%) completed the study (Figure 1).

All subjects were white. Demographic characteristics were balanced between both treatment groups (Table 1). At baseline, 86.0% of FKS518 and 86.8% of reference denosumab recipients were in the WT category >70 kg.

**Table 1:** Subject demographics at baseline; safety analysis set.

**Demographic**	**FKS518** **(*N* = 107)**	**Reference denosumab** **(*N* = 106)**	**Overall** **(*N* = 213)**
**Male, *n* (%)**	107 (100)	106 (100)	213 (100)
**White, not Hispanic or Latino, *n* (%)**	107 (100)	106 (100)	213 (100)
**Mean (SD) age, years**	38.9 (7.1)	38.8 (5.9)	38.8 (6.5)
**Mean (SD) WT, kg**	84.2 (12.1)	83.5 (11.6)	83.8 (11.8)
**50 ≤ WT ≤ 70 kg, *n* (%)**	15 (14.0)	14 (13.2)	29 (13.6)
**70 < WT ≤ 110 kg, *n* (%)**	92 (86.0)	92 (86.8)	184 (86.4)
**Mean (SD) height, cm**	179.8 (6.9)	179.2 (6.4)	179.5 (6.6)
**Mean (SD) BMI**	26.0 (3.0)	26.0 (3.2)	26.0 (3.1)

Abbreviation: WT, weight.

### Pharmacokinetic endpoints

**Figure 2:**
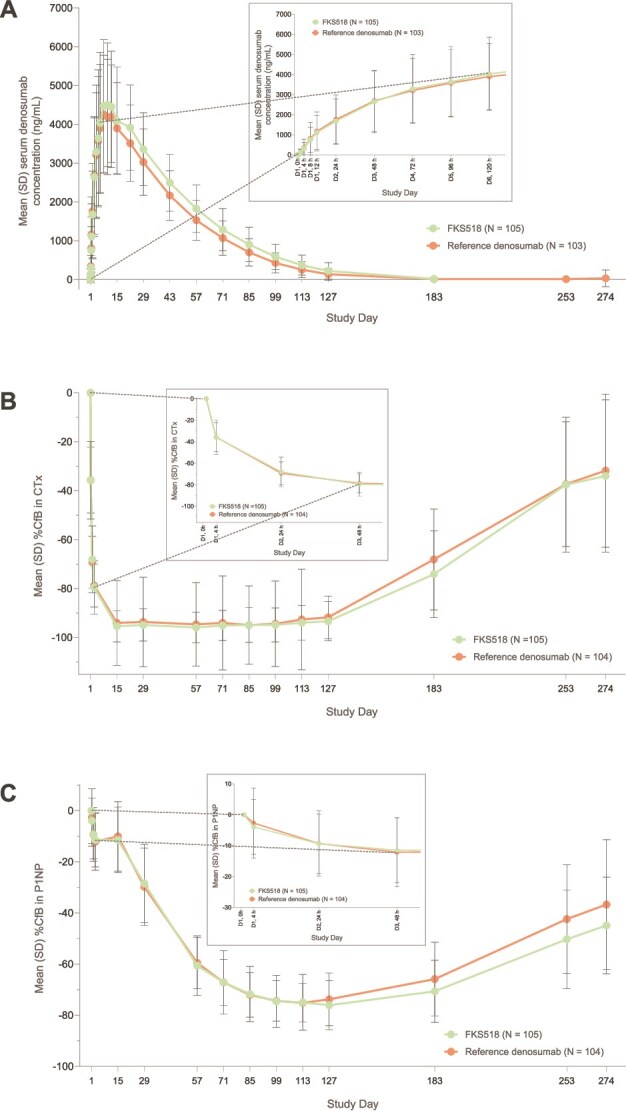
(A) Denosumab serum concentrations over time—PK analysis set, percentage change from baseline in (B) CTx and (C) P1NP levels over time—PD analysis set. Abbreviations: %CfB, percentage change from baseline; D, day; PD, pharmacodynamic; PK, pharmacokinetic.

Following a single s.c. dose of denosumab 60 mg as FKS518 or reference denosumab, quantifiable denosumab concentrations were detected from 1 h postdose for some subjects in both groups, and for all subjects in the FKS518 and reference denosumab groups after 8 and 4 h, respectively. The overall shape of the serum concentration-time profiles of denosumab was similar between the 2 treatments (Figure 2A).

**Figure 3:**
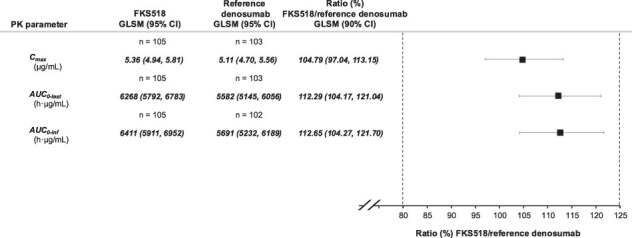
Primary PK analysis to assess the bioequivalence of FKS518 vs reference denosumab—PK analysis set. Abbreviations: AUC_0-inf_, area under the concentration-time curve from time zero to infinity; AUC_0-last_, area under the concentration-time curve from time zero to the last quantifiable concentration; *C*_max_, maximum observed serum concentration; GLSM, geometric least-squares mean; PK, pharmacokinetic.

The 90% CIs for the geometric LS means ratio FKS518/reference denosumab were fully contained within the predefined bioequivalence limits of 80.00%-125.00% for the 3 primary PK parameters (AUC_0-inf_, AUC_0-last_, and *C*_max_; Figure 3). Therefore, PK equivalence between FKS518 and reference denosumab was demonstrated. The percentage of AUC extrapolated beyond *C*_last_ was <10% for all subjects across both treatment groups. Secondary PK parameters were similar between the FKS518 and reference denosumab groups. Median *t*_max_ of denosumab was similar between FKS518 (217.45 h) and reference denosumab (216.03 h). The mean (SD) *t*_1/2_ was 428 (178) h vs 391 (188) h, mean (SD) Vz/F was 6.66 (2.67) L vs 6.83 (2.59) L, and mean (SD) CL/F was 0.0120 (0.0058) L/h vs 0.0135 (0.0057) L/h.

Pharmacokinetic parameters were similar between treatments across body WT categories (50 ≤ WT ≤ 70 kg and 70 < WT ≤ 110 kg).

### Pharmacodynamic endpoints

Following a single s.c. dose of denosumab 60 mg as FKS518 or reference denosumab, serum levels of CTx were reduced for all subjects at 24 h postdose (the second postdose sample) and P1NP levels were reduced for all subjects by day 57 (1344 h postdose). The overall shape of the %CfB-time profiles of CTx and P1NP were similar between FKS518 and reference denosumab (Figure 2B and C), as were the mean (SD) maximum %CfB in serum CTx (97.83 [4.40]% vs 98.81 [3.48]%) and P1NP (78.82 [8.35]% vs 77.47 [9.57]%). The mean (SD) AUEC_0-W40_ for %CfB in CTx (509 604 [63545] h∙% vs 497 021 [71227] h∙%) and mean (SD) AUEC_0-W40_ for %CfB in P1NP (385 222 [65122] h∙% vs 365 876 [62954] h∙%) were also similar between the FKS518 and reference denosumab groups.

### Exposure-response relationship

For both treatments, maximal CTx inhibition occurred at the same time as the denosumab maximum concentrations, at 2 wk postdose. This inhibition remained constant at a close to maximal inhibition from weeks 3 to 19, while denosumab concentrations decreased below the lower limit of quantification (LLOQ) at week 19. For P1NP, there was a lagged effect for both treatments, with maximal P1NP inhibition at week 17, when the denosumab concentrations were nearly below the LLOQ. Mean serum CTx and P1NP levels began to increase from week 19 onwards, although neither had completely returned to baseline levels by the end of study (week 40) (Figure 2).

### Tolerability and safety

A total of 436 TEAEs were reported in 166 (77.9%) subjects; 225 TEAEs in 84 (78.5%) subjects in the FKS518 group and 211 TEAEs in 82 (77.4%) subjects in the reference denosumab group (Table 2). The safety profile of a single s.c. injection of denosumab 60 mg was similar in healthy male subjects who received FKS518 and reference denosumab, and there was no distinguishing pattern in terms of nature, frequency, severity, or resolution of TEAEs across treatments. The most reported TEAEs are summarized in Table 2.

**Table 2:** Treatment-emergent adverse events and local tolerability; safety analysis set.

**Subjects with at least 1**	**FKS518** **(*N* = 107)** ***n* (%) E**	**Reference denosumab** **(*N* = 106)** ***n* (%) E**	**Overall** **(*N* = 213)** ***n* (%) E**
**TEAE**	84 (78.5) 225	82 (77.4) 211	166 (77.9) 436
**Mild TEAE**	30 (28.0) 132	28 (26.4) 117	58 (27.2) 249
**Moderate TEAE**	50 (46.7) 88	50 (47.2) 88	100 (46.9) 176
**Severe TEAE**	3 (2.8) 4	4 (3.8) 6	7 (3.3) 10
**Life-threatening TEAE**	1 (0.9) 1	0	1 (0.5) 1
**Death**	0	0	0
**TR-TEAE**	0	0	0
**SAE**	14 (13.1) 14	9 (8.5) 9	23 (10.8) 23
**AESI**	1 (0.9) 1	0	1 (0.5) 1
**TEAE leading to study discontinuation**	1 (0.9) 1	0	1 (0.5) 1
**Most common TEAE** ^a^			
**Nasopharyngitis**	38 (35.5) 52	47 (44.3) 61	85 (39.9) 113
**Headache**	24 (22.4) 48	15 (14.2) 22	39 (18.3) 70
**COVID-19**	10 (9.3) 10	9 (8.5) 9	19 (8.9) 19
**Back pain**	10 (9.3) 10	6 (5.7) 11	16 (7.5) 21
**Oropharyngeal pain**	5 (4.7) 5	6 (5.7) 6	11 (5.2) 11
**Rhinorrhoea**	5 (4.7) 5	6 (5.7) 6	11 (5.2) 11
**Upper respiratory tract infection**	8 (7.5) 9	2 (1.9) 2	10 (4.7) 11
**Local tolerability**			
**Injection-site reaction**	1 (0.9) 1	6 (5.7) 6	7 (3.3) 7

^a^TEAE by preferred term occurring in at least 5% of either treatment group.

Adverse events were coded using MedDRA Version 24.0.

Subjects are counted only once under the category of their worst grade of TEAE.

Abbreviations: %, percentage of subjects with event; AESI, adverse event of special interest; E, number of events experienced; *n*, number of subjects with event; SAE, serious adverse event; TEAE, treatment-emergent adverse event; TR, treatment-related.

In both groups, the majority of TEAEs were mild or moderate; only 7 (3.3%) study subjects experienced at least 1 severe (Grade 3) TEAE. One subject had a life-threatening (Grade 4) event (suicide attempt). No reported TEAE was considered by the Investigator to be treatment-related. No deaths were reported during this study. Serious TEAEs were reported by 23 subjects; most were COVID-19 infections (*n* = 21), which were considered as an “other medially important event,” regardless of meeting other seriousness criteria. In terms of non-COVID-19 SAEs, 1 patient had bile duct adenocarcinoma (leading to study discontinuation and therefore this was also an AESI) and 1 subject had the life-threatening event of suicide attempt. No other protocol-prespecified AESIs were reported. Few ISR were reported (Table 2), and all were mild injection site bruising. There were no notable differences between FKS518 and reference denosumab in terms of abnormal clinical laboratory parameters or clinically significant vital signs or ECG abnormalities.

### Immunogenicity

All subjects were negative for ADA before dosing and none of the subjects developed ADAs after administration of FKS518 or reference denosumab.

## Discussion

Denosumab is an effective and safe treatment for patients at risk of fractures due to bone loss and is often administered long-term.^6^ It has demonstrated its ability to reduce vertebral and non-vertebral fracture risk in women with breast cancer receiving hormone therapy, men with prostate cancer receiving androgen deprivation therapy, postmenopausal women, and men with osteoporosis and patients with glucocorticoid-induced osteoporosis.^2–5^ However, the costs and cost-effectiveness of denosumab in comparison with some alternative treatments for osteoporosis (such as bisphosphonates) and in some patient subgroups (patients who are not in older age groups, those without prior fracture experience, those with higher BMD T-scores and patients with fewer risk factors) has been questioned,^18^^,^^19^ and the availability of a less costly biosimilar alternative to the originator product may help to address these concerns. In addition, patient access to biologics, such as denosumab, is affected by cost and is variable between countries, which may affect patient outcomes.^20^ It is estimated that in the US, biosimilars could save up to $54 billion on biologic spending from 2017 to 2026.^21^ Biosimilars can provide lower cost alternatives to the originator product, foster competition, contribute to the financial sustainability of healthcare systems and encourage innovation among reference medicine manufacturers.^22^^,^^23^

The design of the current study allowed a comparative assessment the PK, PD, safety, and immunogenicity properties of the denosumab biosimilar FKS518 and the originator (reference) denosumab in healthy male subjects and was designed according to the guidelines and following the feedback of Health Authorities.^11^^,^^12^^,^^24^^,^^25^ A parallel-group design was the preferred approach considering the long half-life of denosumab (25.4 d in healthy subjects)^1^^,^^8^ and the prolonged sampling period of 40 wk (274 d), which would have made a cross-over design impractically long with an increased potential of inadvertent period effects.

Healthy male subjects were chosen as the study population because they are likely to exhibit less PK variability and are therefore more sensitive for evaluating potential differences in PK properties, compared with patients with potential confounding factors, such as underlying diseases and/or concomitant medications. In addition, due to the long half-life and the observed reproductive toxicity of denosumab in animal studies,^1^^,^^8^ study participation for women would have required a long period of restriction on pregnancy. Including only healthy male and no female subjects also further reduced potential variability due to different physiological conditions during the menstrual cycle. Age, gender, race/ethnicity, disease, or disease state do not significantly affect the PK properties of denosumab,^1^^,^^10^ and the PK characteristics of denosumab in healthy subjects and in the approved patient populations are similar. Consequently, the restrictions imposed on the study population are unlikely to influence the generalizability of the key PK study findings to target populations. Randomization was stratified by WT to minimize variability related to differences in body WT, which is considered one of the most influential factors of variability in monoclonal antibody clearance and volume of distribution.^26^ In agreement with feedback from regulatory agencies, the selected dose was the approved dose of the reference denosumab product: 60 mg by s.c. administration,^1^^,^^8^ with no dose modification allowed.

The study demonstrated PK equivalence between FKS518 and reference denosumab after a single s.c. injection. In addition, secondary PK endpoints and PD (bone marker) endpoints were similar between FKS518 and reference denosumab. There were no notable differences between the 2 denosumab products in terms of the frequency, severity, or resolution of TEAEs, SAEs, and AESIs. The number of ISRs was low and balanced, and there was an absence of hypersensitivity TEAEs that were considered severe, serious, or drug related; no anaphylactic reactions occurred in this study. There was no distinguishable pattern between treatments in terms of clinical laboratory results and no clinically meaningful findings were noted in vital signs, ECG, or physical examination assessments, further confirming the overall safety similarity of FKS518 and reference denosumab. No new safety concerns were identified. Furthermore, no patient showed ADA positivity in either treatment group.

The time-course of denosumab concentrations, as well as of CTx and P1NP responses, were similar for the FKS518- and reference denosumab-treated groups. However, as would be expected, maximal CTx inhibition was observed at approximately the same time as maximum denosumab concentrations, whereas inhibition of P1NP was delayed. During bone resorption, osteoclasts secrete proteolytic enzymes that degrade type I collagen into smaller fragments, including CTx. Denosumab binds to RANKL preventing it from interacting with the RANK receptor on osteoclasts and osteoclast precursors, thereby blocking osteoclast differentiation, activation, and survival.^27^ As a result, bone resorption is rapidly suppressed, which is reflected in rapid changes in bone resorption markers, such as CTx.^28^ P1NP is a byproduct of the synthesis of type I collagen, the primary protein in bone matrix, and as such reflects the activity of osteoblasts. Bone formation and bone resorption are interconnected processes within bone remodeling, with osteoblast activation occurring as a physiological response to bone resorption. When denosumab halts osteoclast activity, osteoblasts receive fewer signals to initiate bone formation, and the remodeling cycle is interrupted.^29^ Consequently, P1NP levels decline more slowly, typically over 3-6 mo.^30^^,^^31^

### Conclusions

As for many biologics, originator denosumab is associated with high treatment costs and, in some markets, limited access to treatment. Availability of a denosumab biosimilar is expected to help address these issues. Demonstration of PK equivalence to the originator biologic is a requirement for any biosimilar to be approved for marketing. In this study, PK equivalence was demonstrated between FKS518 and reference denosumab. Secondary PK endpoints, PD (bone biomarker) endpoints, and the safety and immunogenicity profiles of denosumab were similar between FKS518 and reference denosumab. As the current study was 1 of 2 clinical studies included in the clinical development program of FKS518, the results presented here serve as a key contribution to the body of clinical evidence on which FKS518 was established as a biosimilar to originator denosumab.

## Supplementary Material

Supplementary_Material_ziaf176

## Data Availability

The study datasets are not publicly available due to ongoing regulatory filing activities. Once the regulatory application is complete, data will be available from the corresponding author on reasonable request.

## Clinical trial registration

EudraCT Number: 2020-004842-13
