# Supplementary material for: Pharmacokinetics and pharmacodynamics of the denosumab biosimilar FKS518 and reference denosumab in healthy subjects: the Lumiade-1 study
Source: JBMR Plus. 2025 Nov 7;10(1):ziaf176. doi: 10.1093/jbmrpl/ziaf176 (PMC12766911; doi:10.1093/jbmrpl/ziaf176)
Supplement: Supplementary_Material_ziaf176 [file supplementary_material_ziaf176.docx]

# Supplementary material

## Methods

Written informed consent was obtained from each subject prior to performing any screening assessments.

### Pharmacokinetics

Blood sampling to determine denosumab concentrations in serum for pharmacokinetic endpoints were obtained pre-dose (−1 hour) and at 1 hour (± 15 minutes), 2 hours (± 15 minutes), 4 hours (± 30 minutes), 8 hours (± 30 minutes), and 12 hours (± 30 minutes) post dose on day 1, and on days 2 (24 hours± 15 minutes), 3 (48 hours± 1 hour), 4 (72 hours± 1 hour), 5 (96 hours± 1 hour), 6 (120 hours± 3 hours), 8 (168 hours± 3 hours), 10 (216 hours± 8 hours), 12 (264 hours± 8 hours), 15 (336 hours± 8 hours), 22 (504 hours± 8 hours), 29 (672 hours± 1 day), 43 (1008 hours± 2 days), 57 (1344 hours± 2 days), 71 (1680 hours± 2 days), 85 (2016 hours± 2 days), 99 (2352 hours± 2 days), 113 (2688 hours± 5 days), 127 (3024 hours± 6 days), 183 (4368 hours± 7 days), 253 (6048 hours± 7 days), and 274 (6552 hours± 7 days). On days when a PK sample was collected in conjunction with a PD sample, the samples were taken as closely together as possible. On days when a PK sample was collected in conjunction with an immunogenicity sample, the PK sample was collected prior to the immunogenicity sample. The determination of PK concentrations was performed for all subjects in the study using validated methods by a bioanalytical laboratory.

### Pharmacodynamics

Blood samples to determine serum bone biomarker levels (*C*-terminal cross-linking telopeptide of Type 1 collagen [CTx] and procollagen Type 1 N-terminal propeptide [P1NP]) for PK endpoints (to evaluate the association of FKS518/reference denosumab with bone resorption [CTx] and bone formation [P1NP]) were collected at screening (not for baseline), pre-dose, and 4 hours (± 30 minutes; same time as PK sample) post dose on day 1, and on days 2 (24 hours± 15 minutes), 3 (48 hours± 1 hour), 15 (336 hours± 8 hours), 29 (672 hours± 1 day), 57 (1344 hours± 2 days), 71 (1680 hours± 2 days), 85 (2016 hours± 2 days), 99 (2352 hours± 2 days), 113 (2688 hours± 5 days), 127 (3024 hours± 6 days), 183 (4368 hours± 7 days), 253 (6048 hours± 7 days), and 274 (6552 hours± 7 days). Blood samples were collected in the morning (between 8 am and 12 pm), after an overnight fast. On the dosing day, fasting continued until the PD samples at 4 hours (± 30 minutes) after FKS518/reference denosumab administration had been taken. Blood sampling for CTx and P1NP biomarkers was time-matched to the PK sampling to allow the analysis of exposure/response relationship analysis between the percent change from baseline (%CfB) in CTx and P1NP biomarker and denosumab concentrations. In addition, PD parameters for the area under the effect-time curve from week 0 to week 40 (AUEC_0-W40_) for %CfB in serum CTx and %CfB in serum P1NP were derived for comparison of FKS518 and reference denosumab. The %CfB for reduction in bone biomarkers CTx and P1NP was calculated relative to the predose value (baseline) and were expected to be negative values at least up to the time that the drug effect was present. Pre-dose data on day 1 was used as baseline to estimate the AUEC for %CfB; if a baseline value was missing, changes from baseline could not be derived, and the subject was excluded from the PD Analysis Set.

The CTx and P1NP sample analysis was performed for all subjects in the study using validated methods by a qualified laboratory. Bioanalytical reports were generated, one for each CTx and P1NP analysis, by the bioanalytical laboratory.

### Immunogenicity

Sampling for immunogenicity testing was performed during the screening period (for potential assay validation purposes only), on day 1 before dosing and on days 15, 29, 85, 127, 183 and 274 after the PK sample had been collected. Separate blood samples were collected for anti-drug antibody (ADA) and neutralising antibody assessments.

To evaluate the immune response against the tested drugs a multi-tiered approach was employed. A validated stepwise electro-chemiluminescent bridging format, incorporating an acid-dissociation pre-treatment step, was used for this purpose. A validated competitive ligand binding assay was used as well to further assess the neutralizing capacity of ADAs, if any.

### Safety

The safety profile of the IP was assessed through the recording, reporting, and analysis of adverse events (AEs) and physical examination findings including vital signs, a 12-lead electrocardiogram tracing, clinical laboratory tests and local tolerability. Vital signs were assessed at each PK sampling time, whereas physical examinations and clinical laboratory tests were assessed slightly less frequently (at screening, day 1 before dosing, and on days 2, 6, 15, 22, 29, 43, 57, 71, 85, 99, 113, 127, 183, 253 and 274 for physical examinations, and at screening, day 1 before dosing, and on days 2, 6, 15, 29, 85, 127, 183, 253 and 274 for clinical laboratory tests).

Subjects were comprehensively assessed for toxicities from the time of giving the informed consent until completion of the study or withdrawal from the study. The investigator or delegate reported all AEs, whether observed by the investigator or site staff, or reported by the participating subject. Dedicated assessments of common and/or clinically important known or potential adverse reactions for reference denosumab were performed and reported, as appropriate. Signs and symptoms suggestive of hypersensitivity as judged by the investigator were captured and reported. The corresponding standardized Medical Dictionary for Regulatory Activities (MedDRA) queries were used to aggregate these reports. Local tolerability at the administration site was assessed and reported before dosing on day 1 and on days 2–6.
